# Role of Perioperative Intravenous Dexamethasone in Management of Post Adenotonsillectomy Morbidity: A Single Blinded Randomised Controlled Study

**DOI:** 10.24248/eahrj.v6i1.677

**Published:** 2022-07

**Authors:** Zephania Saitabau Abraham, Aveline Aloyce Kahinga

**Affiliations:** a Department of Surgery-University of Dodoma, School of Medicine and Dentistry, Dodoma-Tanzania; b Department of Otorhinolaryngology-Muhimbili University of Health and Allied Sciences, Dar es Salaam-Tanzania

## Abstract

**Objective:**

To determine the role of perioperative intravenous dexamethasone in reducing post adenotonsillectomy morbidity in Dar es Salaam, Tanzania.

**Methods:**

A Prospective, randomised, placebo-controlled study was conducted at Ekenywa Specialized Hospital. Fifty patients were randomised to receive three doses of intravenous dexamethasone (13 males and 12 females) or placebo (13 males and 12 females) administered eight hourly for the first 24hours after surgery (1mg/kg). Data were analysed using statistical package for social sciences version 21 and P-value<.05 was considered to be statistically significant.

**Results:**

Intravenous dexamethasone was found to exert significant effects in terms of reducing the severity of some observed postoperative parameters such as pain scores, post-operative nausea and vomiting (PONV), tolerance to oral fluids, discharge from hospital, postoperative hemorrhage, postoperative pain, re-admission and wound healing between the two groups of patients. In this study, dexamethasone did not significantly exert any effect on fever in the first 24 hours after surgery.

**Conclusions:**

Intravenous dexamethasone is an effective and safe method for reducing post adenotonsillectomy morbidity.

## BACKGROUND

Despite adenotonsillectomy being the commonest surgery in otorhinolaryngology practice, it is associated with significant morbidity especially if perioperative preparations are not well set in place.^1-3^ Following adenotonsillectomy, surgical patients tend to adapt a new life pattern characterized by adapting pain whether at rest or during intake of foods or drinks, postoperative nausea and vomiting (PONV) thus impairing their quality of life post-surgery.^4-8^ When patients experiences pain post-surgery, it tends to limit their intake of oral foods or drinks and may prolong hospital stay.^1,4,6,9-11^

To improve the quality of life during peritonsillectomy by minimising the risk of occurrence of complications, dexamethasone has been proposed to be an important perioperative medication to curb such situation.^6,7,12^ There has been a variable practice globally in otorhinolaryngology pertaining the use of steroids in reduction of morbidity post adenotonsillectomy^6,7^ and our country to date has no any existing standard protocol in peritonsillectomy care of patients and this created an urgent call for designing this study.

Perioperative dexamethasone has shown important effect in terms of decreasing postoperative edema at the surgical site and improving oral intake following adenotonsillectomy through its dual antiemetic and anti-inflammatory effects.^2-4,6,12^

Available studies have shown conflicting results on the role of steroids in reducing morbidity post-adenot onsillectomy.^2-4,6,7,11-13^ Some studies including the one which was conducted in Switzerland where a meta-analysis of 17 trials involving use of dexamethasone for prevention of PONV in surgical patients concluded its significance in exerting antiemetic effect compared with placebo.^14^

The aim of this study was to determine the role of perioperative intravenous dexamethasone in reducing post adenotonsillectomy morbidity.

## MATERIALS AND METHODS

### Study Area and Population

The study was conducted at Ekenywa Specialised Hospital from January to June 2020 at the Department of Otorhinolaryngology where patients aged 2 to 18 years who were fit for adenotonsillectomy were recruited. The department of otorhinolaryngology attends about 200 outpatients on daily basis and performs an average of six elective surgeries per day.

### Study Design

A randomised single blinded study design was conducted to evaluate the effect of intravenous dexamethasone in managing and controlling morbidity after adenotonsillectomy in patients aged 2 to 18 years at Ekenywa Specialised Hospital.

### Inclusion Criteria

Selected patients aged 2 to 18 years who were fit for adenotonsillectomy were recruited after obtaining parents' or caretakers' consents.

### Exclusion Criteria

Patients who had contraindications to steroids use and those with bleeding or medical disorders were excluded upon fulfilling routine pre-operative protocols for tonsillectomy/adenotonsillectomy which included history taking, thorough ear, nose and throat examination, and laboratory workup such as full blood picture, blood grouping and bleeding indices (bleeding time, prothrombin/partial thromboplastin time and international normalized ratio).

### Randomisation Technique

Fifty patients between 2 and 18 years of age were randomized into intervention (n=25) or placebo (n=25). Those belonging to intervention group received three doses of intravenous dexamethasone being administered eight hourly for the first 24hours after surgery (1mg/kg). Those belonging to the control group/placebo received 5mls of normal saline. Maximum dose used was 24mg and no adverse effect of this drug was reported in this study. In our study, we administered dexamethasone 1 mg/kg, subject to a maximum dose of 8mg immediately after induction of anesthesia and the anesthetic and surgical techniques were standardized for tonsillectomy and / or adenoidectomy.

Randomization of patients commenced at the operating theatre where those belonging to the steroid arm received 1mg/kg of such drug while those assigned to placebo/saline arm received 5mls of normal saline. All the participants were blinded to treatment allocations but not investigators.

To prevent confounding effect of antiemetic drugs to surgical patients, such medication was not administered. While patients were in the wards post-surgery, trained nurses administered intravenous dexamethasone. Similar analgesics (intravenous paracetamol at 15mg/kg was administered to all surgical patients) and general anaesthetic agents (induction being done using halothane/sevoflurane and being maintained on isoflurane) were administered to cases and controls intraoperatively.

To perform tonsillectomy/adenotonsillectomy, dissection method was utilized and electrocautery was applied only when patients experienced persistent bleeding from the surgical sites intraoperatively. Patients were then kept for observation for 24hours post-surgery and thereafter discharged ready for further postoperative follow-ups scheduled on the 7^th^, 14^th^, 21^st^ and 28^th^ day.

### Statistical Analysis

Statistical package for social sciences (SPSS) version 21from the University of Sussex in England was utilised to analyze data. Chi-square and Fisher's exact tests were used to establish the relationship between variables in both the study and control groups. A *p*-value<.05 was considered to be statistically significant.

### Ethical Considerations

Parents or caretakers provided written informed consent since the study participants were aged 2 to 18 years. The study procedures and drug administration were explained to parents and caretakers. Ethical clearance was granted on 10^th^ December 2019 with number Ref: *ESH/2019/05*. Study participants were not declined from receiving medical care even upon refusing from participating in the study. Confidentiality was guaranteed as no names or identifiers of the participants were collected during data collection.

## RESULTS

### Fisher's Exact Test

Patients in the placebo group were more likely to experience PONV, pain, fever, delayed healing of the tonsillar fossa, postoperative hemorrhage and prolonged hospital stay (Table 1). All patients receiving dexamethasone were able to tolerate 400mls of oral fluids at 8 hours after surgery whereas, none of the patients receiving the placebo could tolerate oral fluids at 8 hours postoperatively (Table 1). All patients receiving dexamethasone were able to resume to routine oral feeds earlier than their counterparts in the placebo group (Table 1). None of the patients from dexamethasone group was re-admitted signifying no any complications post adenotonsillectomy. But none in the control group completed healing on seventh postoperative day. (Table 1).

**TABLE 1:** Comparison of Post-Adenotonsillectomy Morbidities in the Two Groups

variables N=25	Placebo group N=25 n (%)	Dexamethasone group N=25 n (%)	Odds ratio (95% CI)	P-value
PONV	16 (64)	1 (4)	0.03 (0.0–0.13)	<0.001
Pain	11 (44)	3 (12)	0.19 (0.06–0.54)	<0.001^a^
Fever	5 (20)	1 (4)	0.18 (0.04–0.95)	<0.04^a^
Delayed healing of the tonsillar fossa	25 (100)	2 (8)	0 (0.00–0.01)	<0.001
Intolerance to oral fluids post-surgery	25 (100)	0 (0.0)	0 (0.00–0.002)	<0.001
Delayed tendency to resume to routine oral feeds	5 (20)	0 (0.0)	0 (0.00–0.001)	<0.001
Re-admissions,	4 (16)	0 (0.0)	0 (0.00–0.001)	<0.001
Prolonged hospital stay,	1 (4)	0 (0.0)	0 (0.00–0.001)	<0.001
Postoperative hemorrhage,	1 (4)	0 (0.0)	0 (0.00–0.002)	<0.001

a Fisher's Exact Test

## DISCUSSION

Despite adenotonsillectomy being the commonly performed procedure it is associated with postoperative morbidity. There has been no any study in our country that has tried to explore the role of intravenous dexamethasone in reducing postoperative morbidity. The aim of this study was to determine the role of perioperative intravenous dexamethasone in reducing post adenotonsillectomy morbidity at Ekenywa Specialised Hospital in Dar es Salaam, Tanzania.

Adenotonsillectomy which is the commonest surgical procedure in otorhinolaryngology causes tissue injury induced acute inflammation, nerve irritation and spasm of exposed pharyngeal muscles which consequently play a role in the genesis of post adenotonsillectomy pain.^3-5,9-12^

There has been no consensus regarding the use of intravenous corticosteroids for adenotonsillectomy from the available literatures since the available studies have either ended up lacking the control group or lacking standardization for the surgical techniques and also there are conflicting ideas about the type and dose of the suitable corticosteroid, whether to use single or multiple doses and whether to use alone or as an adjuvant to other drugs.^1,2,4-6,10,11^

The main objective of this study was to find the effect of steroids on reducing the post-adenotonsillectomy morbidity (such as pain, edema, nausea and vomiting, delayed tendency of resuming to normal oral feeds). Dexamethasone was selected as an ideal perioperative steroid because it has a long half-life of 36 to 48 hours and has glucocorticoid activity.^4,6,7,10-12^

Postoperatively, those patients belonging to dexamethasone group were kept on intravenous dexamethasone for the first 24 hours after surgery. The administered dose conformed with what was established in other studies where the dosage of dexamethasone, ranging from 0.15 mg/kg to 1 mg/kg with maximum doses ranging from 8 to 25 mg have been commonly used in children with safety.^7,9^ In a large study involving 133 patients, Splinter and Roberts have used 0.15 mg/kg dexamethasone with good results.^15^

In our study, majority of dexamethasone treated patients did not require extra analgesics by the time they were discharged 24 hours after surgery. This indicates that dexamethasone is an effective analgesic for patients who have undergone adenotonsillectomy. This finding is in line with what was found elsewhere.^6,12^ The current study reports few frequencies of PONV occurrence, thus supporting the hypothesis that intravenous dexamethasone is effective in managing and controlling the occurrence of PONV post-surgery. This finding is similar to what was found by Pappas et al who found a 40-60% decrease in the incidence of PONV after intravenous dexamethasone.^16^

The steroids administered to patients in the intervention group was effective in reducing edema post adenotonsillectomy. At the end of 24 hours after surgery, the incidence of edema of soft palate and uvula were significantly less in the dexamethasone treated patients compared to controls. The marked decrease in edema had positive impact in terms of improving oral intake of foods post-surgery due to less inflammation and pain. Such finding correlate with what was found by Steward et al in meta-analysis where children who received dexamethasone were more likely to resume soft or solid diet 24 hours post tonsillectomy while none from the control group (received saline) was able to tolerate oral feeds at 8-hours post-surgery.^17^

Findings in this study revealed that intravenous dexamethasone was not useful in controlling fever post adenotonsillectomy, a finding similar to the studies which were conducted in Kuwait and United States of America.^12,18^

In terms of duration of hospital stay post adenotonsillectomy, all patients were fit for discharge 24 hours post adenotonsillectomy and this may be due to reduced pain and inflammation and thus reduced overall morbidity. Only one patient from the placebo group had prolonged hospital stay due to severe dysphagia, fever and edema of the uvula. Such finding appears to be similar to what was found in a study that was conducted in Kuwait.^12^ but different from what was established in a study that was conducted in the United States of America where there was no difference in length of hospital stay between dexamethasone and control groups.^18^

Pertaining, re-admission post adenotonsillectomy, none of the study group was re-admitted whereas four patients from the control group were re-admitted (one patient with secondary hemorrhage and three patients with severe dysphagia, fever and pain post-surgery). Such finding appears to be similar to what was established in a study which was conducted in Kuwait.^12^

During routine follow up of patients after surgery, twenty-three patients from dexamethasone group completed healing with normal tonsillar bed on seventh day postoperatively and none in the control group completed healing on seventh day postoperatively and similarly, only 20 patients from the control group completed healing after the fourteenth day postoperatively. Such similarity in terms of non-delayed wound healing following perioperative dexamethasone appear similar to what was found in a study which was conducted in Kuwait.^12^ Since none in the placebo group completed healing on the seventh postoperatively, steroids have significantly promoted healing in post-adenotonsillectomy patients.

## CONCLUSION

This study has established that routine use of perioperative intravenous dexamethasone in patients undergoing adenotonsillectomy can significantly decrease the incidence of morbidity such as reduction of PONV, pain, and edema of uvula and soft palate, improves oral intake, reduces incidence of readmission, and promotes early healing of the tonsillar bed. Intravenous dexamethasone given three times daily is safe though did not show an impact in reduction of postoperative pyrexia.

### Recommendations

There is an urgent need for Ekenywa hospital to establish standardized peritonsillectomy protocol of using intravenous dexamethasone due to its role in reducing morbidity post adenotonsillectomy.
